# Cell surface Toll-like receptor polymorphisms influence *Bartonella* and ectoparasite infections in striped hamsters

**DOI:** 10.1016/j.isci.2025.112883

**Published:** 2025-06-13

**Authors:** Xinchang Lun, Yujuan Yue, Yiguan Wang, Guichang Li, Ning Zhao, Fengxia Meng, Qiyong Liu, Pengbo Liu, Zihao Wang, Zhenxu Wang, Xiuping Song, Jun Wang, Ying Liang, Liang Lu

**Affiliations:** 1 National Key Laboratory of Intelligent Tracking and Forecasting for Infectious Diseases, National Institute for Communicable Disease Control and Prevention, Chinese Center for Disease Control and Prevention, Beijing, China; 2 CAS Key Laboratory of Insect Developmental and Evolutionary Biology, CAS Center for Excellence in Molecular Plant Sciences, Shanghai, China; 3 School of Public Health, Nanjing Medical University, Nanjing, China

**Keywords:** Microbiology, Cell biology

## Abstract

This study explored how polymorphisms in cell surface Toll-like receptors (TLRs) influence susceptibility to *Bartonella* and ectoparasite infections in striped hamsters. Among six TLR genes, four sites in TLR1, TLR4, and TLR10 genes were associated with *Bartonella* susceptibility, while twelve sites across TLR4, TLR5, and TLR10 contributed to flea resistance. Similarly, eleven sites in TLR5, TLR6, and TLR10 were linked to gamasid mite parasitism. Genetic polymorphism analysis revealed that when heterozygous or rare genotypes protected the host from infections, the polymorphisms of these sites in uninfected individuals exceeded that of the infected group. The distribution of these sites on the three-dimensional structure of TLRs varied. All *Bartonella*-related sites were in the extracellular domain, whereas some of those related to fleas and gamasid mites were located in other domains. This research highlights the importance of cell surface TLRs in immune regulation and provides insights into evolutionary dynamics in natural environments.

## Highlights

• TLR gene polymorphisms were associated with *Bartonella*, flea, and mite infections in hamsters
• TLR polymorphisms differentially influenced susceptibility to distinct pathogens
• TLR sites linked to distinct pathogens varied in spatial localization
• The study uncovered TLR-driven immunity and host adaptation in wild hamsters

## Graphical abstract

**Figure:**
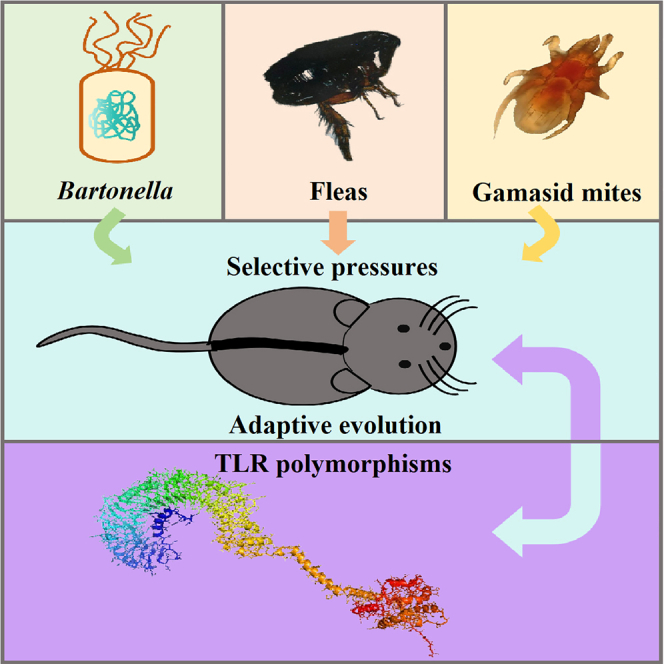

## Introduction

The striped hamster (*Cricetulus barabensis*), a small mammal, is widely distributed in the temperate zone of East Asia,^1^ with high reproductive ability.^2^^,^^3^ It plays a vital role in maintaining ecological balance and biodiversity as an essential component of ecosystems.^4^ However, the populations of this species are vulnerable to various pathogens and parasites in their natural habitats.^5^^,^^6^^,^^7^ Pathogens and vectors exert pressure on their vertebrate hosts in multiple ways, driving the selection of different host phenotypes.^8^ The dynamic interaction between rodents, pathogens, and parasites is an ongoing process of co-evolution, which significantly influences biological phenomena such as local adaptation, species formation, and parasite resistance.^9^ Pathogens and parasites exert strong selective pressure on their hosts by infecting and parasitizing them, which can trigger genetic evolution in host populations, promoting resistance or adaptation to these invasions. At the same time, pathogens and parasites evolve new strategies to overcome the host’s defense mechanisms. The host’s resistance to infection stems from its co-evolutionary process with parasites, in which the host’s genotype and the parasite’s genotype play important roles. Over time, this dynamic interaction continues to drive the adaptive evolution of both parties, affecting the host’s ability to defend against infections and the parasite’s immune escape strategy.^10^ The co-evolution or “arms race” between hosts and parasites is a key factor that shapes population genetic diversity and can drive rapid genomic and phenotypic differentiation, potentially leading to speciation.^9^^,^^11^

The co-evolution between hosts and parasitic organisms involves both the innate and adaptive immune systems.^12^^,^^13^ Specifically, the innate immune system serves as the first line of defense, recognizing and responding to pathogens through pattern recognition receptors (PRRs), which distinguish microbial invaders from the host’s cells.^14^^,^^15^ PRRs play a crucial role in initiating or regulating immune responses, recognizing conserved pathogen-associated molecular patterns (PAMPs).^16^^,^^17^^,^^18^ Among PRRs, Toll-like receptors (TLRs) are particularly important for immune regulation.^19^^,^^20^^,^^21^ TLRs can be divided into two categories based on their locations, functions and ligand types they recognize: cell surface TLRs (TLR1, TLR2, TLR4, TLR5, TLR6, and TLR10) are expressed on the cell surface and mainly recognize microbial membrane components such as lipids, lipoproteins and proteins, while intracellular TLRs (TLR3, TLR7, TLR8, and TLR9) are expressed in intracellular vesicles such as the endosomes, and lysosomes to recognize microbial nucleic acids.^22^^,^^23^^,^^24^ Multiple studies have shown significant associations between TLR gene polymorphisms and pathogen/parasite infections.^25^^,^^26^^,^^27^^,^^28^ Meanwhile, cell surface TLRs are most likely to participate in the induction of inflammatory responses^29^ and TLR gene polymorphisms significantly influence susceptibility and inflammatory responses to infections.^30^

Research has shown that rodents are considered one of the main hosts of *Bartonella*, and among the reported species of *Bartonella*, about two-thirds of them are hosted by rodents.^31^ The *Bartonella* species of rodents are more diverse than other animals besides bats.^32^ *Bartonella* infection is commonly vectored by fleas, ticks, mites, and lice, as well as through scratches from infected animals.^33^^,^^34^ Additionally, the feces of infected arthropods can spread *Bartonella* to humans and other mammals through skin surface lesions.^35^ TLR polymorphisms in hosts have been associated with *Bartonella* infections, emphasizing the important role of TLRs in immune responses. For example, in free-living bank voles (*Myodes glareolus*), polymorphisms of TLR1 and TLR5 were found to be associated with the risk of *Bartonella* infections,^36^ while TLR2 polymorphisms were linked to *Bartonella* susceptibility in brown rats. ^37^

In this study, we analyzed the polymorphisms in six cell surface TLR genes (TLR1, TLR2, TLR4, TLR5, TLR6, and TLR10) in striped hamsters, aiming to determine their association with *Bartonella* infections and ectoparasite parasitism, including fleas and gamasid mites. By analyzing the genetic variations in these TLR genes, we sought to shed light on the genetic evolution of TLRs in hosts exposed to pathogen infections and ectoparasite challenges. Furthermore, we explored the potential trade-offs associated with the advantages and disadvantages of TLR polymorphisms in hosts that are continually confronted with pathogens and ectoparasites. This research aims to offer valuable insights into the evolutionary and ecological dynamics of host-parasite-pathogen interactions.

## Results

### *Bartonella* infections linked to gamasid mites, not fleas

**Figure 1:**
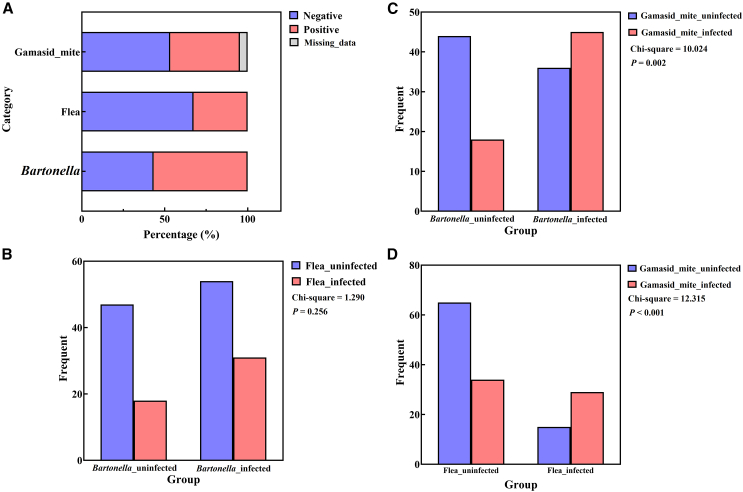
Analysis of *Bartonella* and ectoparasite infection rates in striped hamsters (A) Infection rates of *Bartonella* and ectoparasites in striped hamsters. (B) Differential analysis between *Bartonella* infections and flea parasitism in striped hamsters. (C) Differential analysis between *Bartonella* infections and gamasid mite parasitism. (D) Differential analysis between flea parasitism and gamasid mite parasitism in striped hamsters.

A total of 150 striped hamsters were collected from six locations in Inner Mongolia, 2021–2022. Among them, 85 were infected with *Bartonella*, 49 with fleas, and 63 with gamasid mites (Figure 1A). We compared the prevalence of flea infections between hamsters with and without *Bartonella* infections. The results indicated no significant difference in flea parasitism between the two groups, suggesting the *Bartonella* infection is independent of flea infections in striped hamsters. However, a significant difference was observed in gamasid mite parasitism between hamsters with and without *Bartonella* infections - the group with *Bartonella* infections had a higher number of hamsters parasitized by gamasid mites. This finding may suggest a potential role for gamasid mites in *Bartonella* infections. Additionally, there was a significant difference in gamasid mite parasitism between striped hamsters with and without flea parasitism, indicating a possible “symbiotic” relationship among different species of ectoparasites (Figures 1B–1D).

### Analysis of genetic diversity in cell surface Toll-like receptor genes

**Figure 2:**
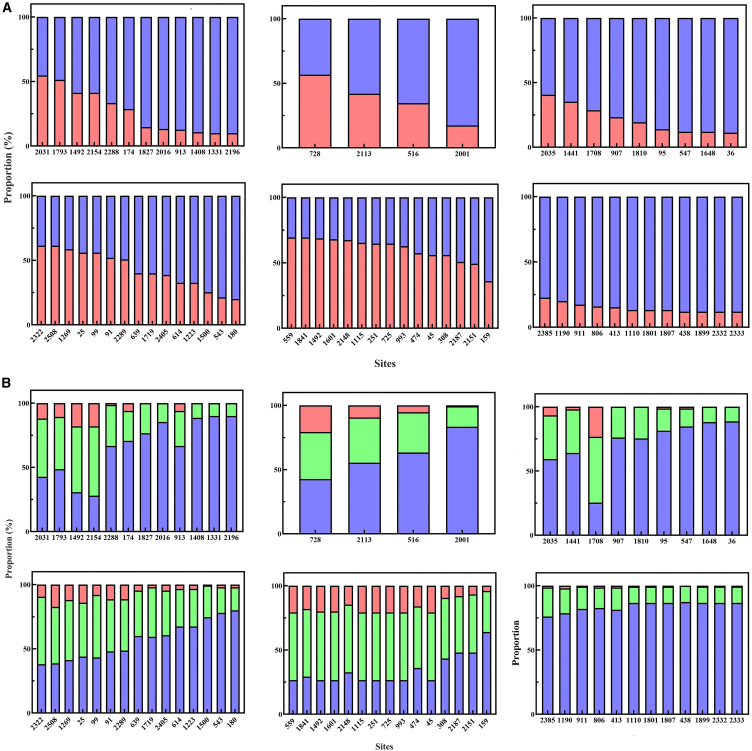
Genotypic statistics of cell surface TLR genes screening sites (A) Genotype proportions of selected sites in TLR1, 2, 4, 5, 6, and 10 genes obtained through sequence alignment. Blue represents the proportion of common genotypes, while red represents the proportion of heterozygous genotypes and rare genotypes. (B) Genotype proportions of selected sites in TLR1, 2, 4, 5, 6, and 10 genes obtained through peak plot comparison. Blue represents the proportion of common genotypes, green represents the proportion of heterozygous genotypes, and red represents the proportion of rare genotypes.

The longest coding sequence (CDS) fragments in the six cell surface TLR genes varied from 2260 bp to 2580 bp, with the proportion of polymorphic sites ranging between 1.68% for TLR4 and 3.49% for TLR5 (Table 1). The sequence alignment analysis revealed the presence of 12, 4, 9, and 12 polymorphic sites on the TLR1, TLR2, TLR4, and TLR10 genes, respectively, with over 10% of samples at each site exhibiting base mutations. Notably, the TLR5 and TLR6 genes exhibited a higher number of polymorphic sites, with over 10% of samples showing base mutations within the same site. For subsequent analyses, only polymorphic sites that were among the top 10% of the sample size and had the highest number of base mutation samples were included (Figure 2A). The peak plot indicated that the common genotype (high-frequency homozygous genotype), heterozygous genotype, and rare genotype (low-frequency homozygous genotype) of the three groups of sites were completely consistent across each sample. These sites correspond to positions 559, 1115, 251, 725, 93, and 45 on the TLR6 gene; Positions 1492 and 1601 on the TLR6 gene; and positions 1110, 1801, 1807, 1899, 2332, and 2333 on the TLR10 gene (Figure 2B). To ensure the accuracy of the model, we eliminated duplicate variables. Consequently, only positions 559 and 1492 of the TLR6 gene, along with position 1110 of the TLR10 gene were selected to represent the above sites as representative inputs for random forest (RF) analysis, Boruta algorithm analysis, and gradient boosting machine (GBM) analysis (Figure 2B).

**Table 1:** Statistical analysis of polymorphic sites in cell surface TLR genes

Gene	Length	Number of polymorphic sites	Proportion (%)
TLR1	2388	62	2.60
TLR2	2370	49	2.07
TLR4	2260	38	1.68
TLR5	2580	90	3.49
TLR6	2451	58	2.37
TLR10	2430	83	3.42

### Gender and weight influence ectoparasites, but not *Bartonella* infections

**Figure 3:**
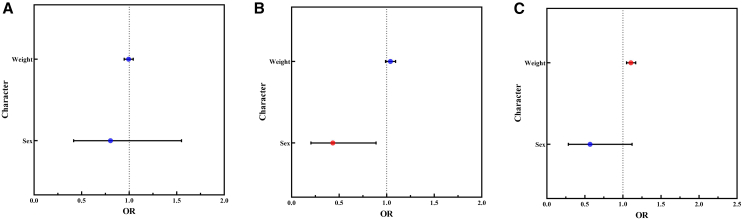
The influence of gender and body weight on *Bartonella* and ectoparasite infections in striped hamsters The influence of gender and body weight on the infections of (A) *Bartonella*, (B) fleas, and (C) gamasid mites, respectively.

Through generalized linear model (GLM) analysis, we found that the gender and weight of striped hamsters did not affect *Bartonella* infections but had a significant effect on ectoparasite infections. Specifically, female hamsters were less likely to carry fleas compared to males. Additionally, heavier hamsters had a higher risk of carrying gamasid mites (Figure 3). Consequently, we included gender and body weight in our corresponding models.

### Genetic variants in Toll-like receptor genes influence *Bartonella* and ectoparasite infections

We compared the genotype frequencies between *Bartonella*-infected and uninfected striped hamsters, as well as between those with and without fleas or gamasid mite infestations. We identified 8, 13, and 14 sites with different genotype frequencies in the *Bartonella*-infected, flea-infected, and gamasid mite-infected groups, respectively (Tables S2–S4). These genotypes likely influence the risk of *Bartonella* infection and ectoparasite parasitism.

**Figure 4:**
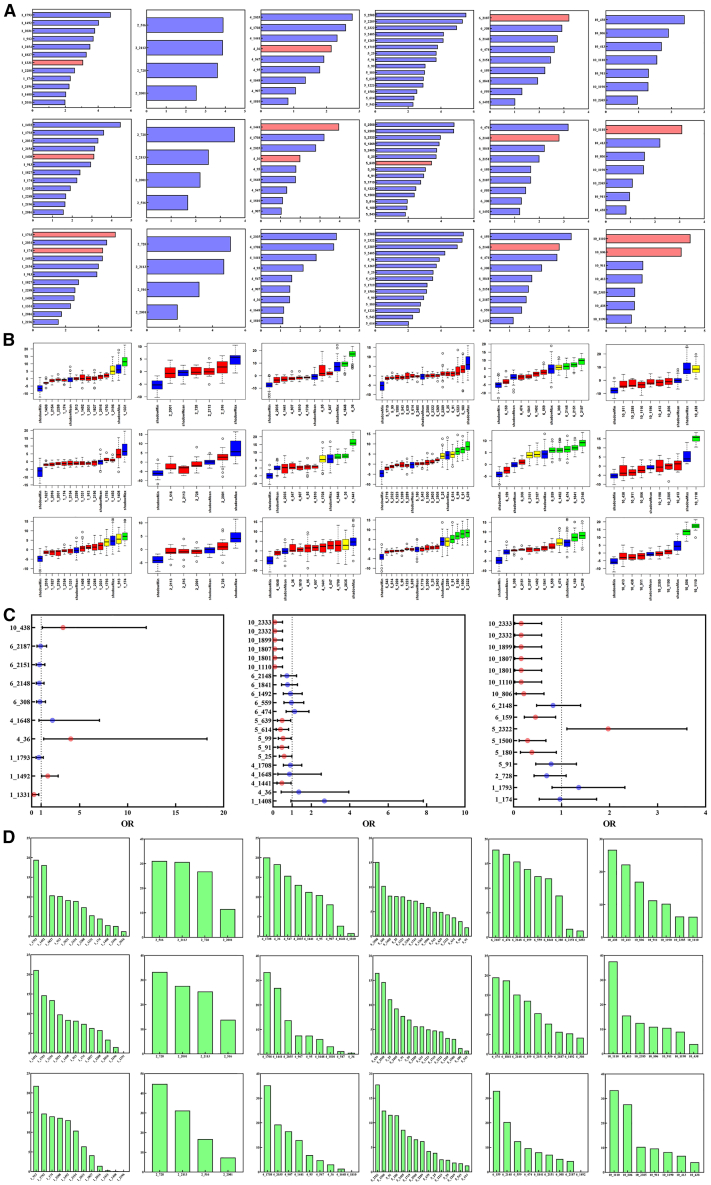
The relationship between exogenous organism infections and cell surface TLR gene polymorphisms (A) Importance scores of polymorphisms at selected sites of TLR1, 2, 4, 5, 6, and 10 genes in *Bartonella*, flea, and gamasid mite infections based on RF analysis. The vertical axis represents the sites, while the horizontal axis represents the Mean Decrease Gini. Blue represents variables with *p* > 0.05, and red represents variables with *p* < 0.05. (B) Importance of polymorphisms at selected sites of TLR1, 2, 4, 5, 6, and 10 genes for *Bartonella*, flea and gamasid mite infections analyzed by the Boruta algorithm. Blue boxplots represent shadow features, red boxplots represent rejected features, yellow boxplots represent the need for further feature identification, and green boxplots represent confirmed features. (C) Sites associated with *Bartonella*, flea and gamasid mite infections in striped hamsters obtained through three statistical methods, then analyzed individually to determine their effects on *Bartonella*, flea and gamasid mite infections by GLM. (D) Importance ranking of selected sites related to *Bartonella*, flea and gamasid mite infections for TLR1, 2, 4, 5, 6, and 10 genes by GBM model. The vertical axis represents relative influence, and the horizontal axis represents the sites.

Using RF analysis, we calculated the significance scores (mean decrease Gini, MDG) for each selected site of the six cell surface TLR genes regarding infections with *Bartonella*, fleas and gamasid mites. The genotypes at positions 1331 of TLR1, 36 of TLR4, and 2187 of TLR6 significantly affected *Bartonella* infection, with MDGs of 3.08, 2.33, and 3.22, respectively. For flea infections, significant genotypes were found at positions 1408 of TLR1, 1441 and 36 of TLR4, 639 of TLR5, 2148 of TLR6 and 1110 (1801, 1807, 1899, 2332 and 2333) of TLR 10, with MDGs of 3.11, 3.97, 2.00, 3.43, 2.81, and 3.10, respectively. For gamasid mite infections, significant genotypes were observed at positions 1793 and 174 of TLR1, 2148 of TLR6, and 1110 (1801, 1807, 1899, 2332 and 2333) of TLR 10, with MDGs of 5.05, 4.27, 3.53, 4.28, and 3.83, respectively (Figure 4A).

The Boruta algorithm assessed the importance of each polymorphic site in the six TLR genes for *Bartonella*, flea, and gamasid mite infections. High-importance scores were noted for positions 1331 of TLR1, 1648 and 36 of TLR4, 2148, 2151, and 2187 of TLR6 for *Bartonella* infections; Positions 1648, 36 and 1441 of TLR4, 99, 91, and 639 of TLR5, 559 (1115, 251, 725, 93, and 45), 1492 (1601), 474, 1841, and 2148 of TLR6 and 1110 (1801, 1807, 1899, 2332, and 2333) of TLR10 for flea infections; And positions 174 of TLR1, 91, 180, 1500, and 2322 of TLR5, 159 and 2148 of TLR6 and 806 and 1110 (1801, 1807, 1899, 2332, and 2333) of TLR10 for gamasid mite infections (Figure 4B).

Through these analyses, we identified 10 sites in six TLR genes related to *Bartonella* infection, 27 to flea infection, and 16 to gamasid mite infections (Table 2). Univariate analyses using GLM revealed that among the sites significantly correlated with *Bartonella* infections, 3/4 showed positive correlations, indicating that heterozygous or rare genotypes were more susceptible to *Bartonella*. In contrast, all 12 sites significantly correlated with flea parasitism, and 10 out of 11 sites correlated with gamasid mite parasitism showed negative correlations, suggesting heterozygous or rare genotypes were less likely to harbor ectoparasites (Figure 4C).

**Table 2:** Sites of cell surface TLR genes related to exogenous organism infections identified through three statistical methods

	Chi-square test	RF analysis	Boruta algorithm	Total
*Bartonella*	1_1331,1_1492,1_1793,4_36,6_308,6_2151,6_2187,10_438	1_1331,4_36,6_2187	1_1331,4_36,4_1648,6_2148,6_2151,6_2187	1_1331,1_1492,1_1793,4_36,4_1648,6_308,6_2148,6_2151,6_2187,10_438
Fleas	4_1441,4_1708,5_25,5_91,5_99,5_614,5_639,10_1110,10_1801,10_1807,10_1899,10_2332,10_2333	1_1408,4_36,4_1441,5_639,6_2148,10_1110,10_1801,10_1807,10_1899,10_2332,10_2333	4_36,4_1441,4_1648,5_91,5_99,5_639,6_45,6_251,6_474,6_559,6_725,6_993,6_1115,6_1492,6_1601,6_1841,6_2148,10_1110,10_1801,10_1807,10_1899,10_2332,10_2333	1_1408,4_36,4_1441,4_1648,4_1708,5_25,5_91,5_99,5_614,5_639,6_45,6_251,6_474,6_559,6_725,6_993,6_1115,6_1492,6_1601,6_1841,6_2148,10_1110,10_1801,10_1807,10_1899,10_2332,10_2333
Gamasid mites	1_174,2_728,5_91,5_180,5_1500,5_2322,6_159,10_806,10_1110,10_1801,10_1807,10_1899,10_2332,10_2333	1_174,1_1793,6_2148,10_806,10_1110,10_1801,10_1807,10_1899,10_2332,10_2333	1_174,5_91,5_180,5_1500,5_2322,6_159,6_2148,10_806,10_1110,10_1801,10_1807,10_1899,10_2332,10_2333	1_174,1_1793,2_728,5_91,5_180,5_1500,5_2322,6_159,6_2148,10_806,10_1110,10_1801,10_1807,10_1899,10_2332,10_2333

Using the aforementioned methods, we successfully identified sites of cell surface TLR genes that significantly affected *Bartonella* infection, flea, and gamasid mite parasitism in striped hamsters (Figure 3). To further gauge the relative significance of each site concerning these infections, we used the GBM model. Ranked by importance, we found that the importance of these sites was significantly associated with exogenous organism infections (*Bartonella*, flea, and gamasid mite infections), and these sites in their respective genes were mostly ranked high (Figure 4D).

### Genetic diversity linked to infection susceptibility

**Figure 5:**
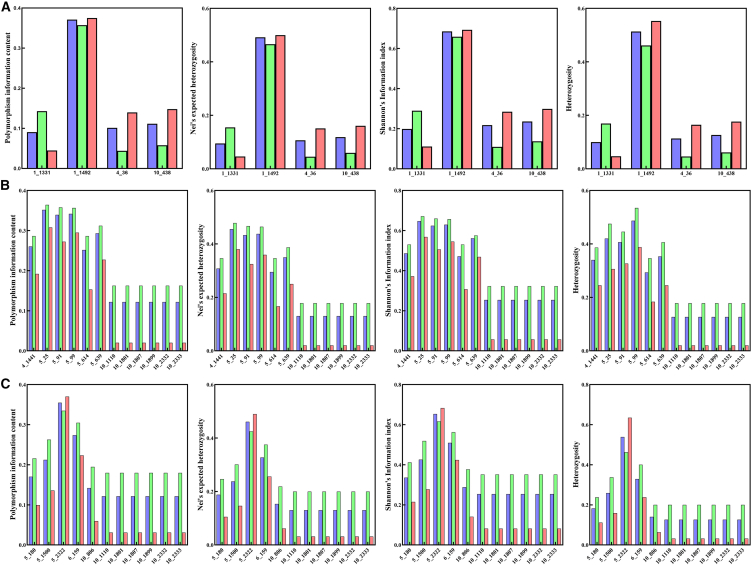
Genetic diversity analysis of identified sites in total, uninfected and infected groups (A) Genetic polymorphisms of identified sites in the total sample group, *Bartonella*-uninfected group, and *Bartonella*-infected group. (B) Genetic polymorphisms of identified sites in the total sample group, flea-uninfected group, and flea-infected group. (C) Genetic polymorphisms of identified sites in the total sample group, gamasid mite-uninfected group, and gamasid mite-infected group. Blue indicators represent the total sample group. Green indicators represent the uninfected group. Red indicators represent the infected group.

Through the analysis of four indicators, including Shannon’s information index (I), Nei’s expected heterozygosity (Nei), heterozygosity, and polymorphic information content (PIC), we obtained genetic polymorphisms in cell surface TLR genes that significantly influence *Bartonella*, flea parasitism, and gamasid mite infections in striped hamsters. This phenomenon was consistent in each comparison group of striped hamsters, regardless of whether they had been infected with *Bartonella*, fleas, or gamasid mites. Specifically, if a heterozygous genotype or rare genotype at this site conferred protection against infection by exogenous organisms, the genetic diversity among uninfected striped hamsters was higher than that of the infected group. Conversely, if heterozygous or rare genotypes at a site were associated with an increased susceptibility to infection, the genetic diversity among uninfected striped hamsters was lower than that of the infected group (Figure 5).

### Spatial distribution of polymorphic sites in Toll-like receptor genes associated with different infections

**Figure 6:**
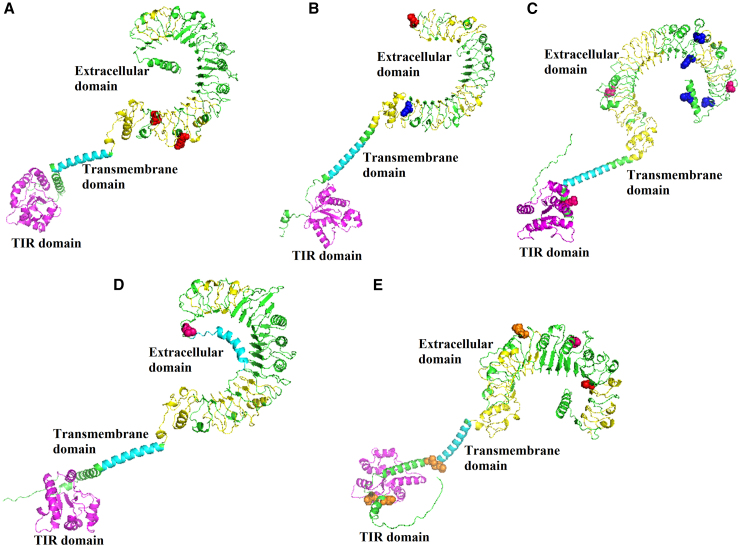
Sites related to exogenous organism infections visualized on the 3D structure of TLRs (A–E) The localization of polymorphic sites associated with exogenous organism infections determined by (A) TLR1, (B) TLR4, (C) TLR5, (D) TLR6, and (E) TLR10, respectively. Red, blue, pink, and orange represent the localization of polymorphic sites associated with *Bartonella* infections, flea parasitism, gamasid mite parasitism, and both flea and gamasid mite parasitism, respectively.

PyMOL software was used to elucidate the spatial distribution of polymorphic sites in TLR gene products by analyzing the three-dimensional (3D) structure of each TLR protein. All four sites associated with *Bartonella* infections were distributed in the extracellular domain. The five sites (positions 1801, 1807, 1899, 2332, and 2333) of TLR10 gene associated with flea parasitism were located either in the transmembrane domain or Toll/interleukin-1 receptor (TIR) domain, while the remaining seven sites (position 1441 of TLR4, positions 25, 91, 99, 614, and 639 of TLR5, position 1110 of TLR10) were distributed in the extracellular domain or signal peptide position. Additionally, more than half of the sites associated with gamasid mite parasitism were distributed in the transmembrane and TIR domains (Figure 6).

## Discussion

This study described the prevalence of *Bartonella* infections and ectoparasite carriage in striped hamsters captured in Inner Mongolia Autonomous Region. It analyzed genetic polymorphisms in the longest CDSs of six cell surface TLR genes and their relationship with *Bartonella* and ectoparasite infections. We identified sites significantly affecting *Bartonella* and ectoparasites infections and explored the genetic diversity and population structure of these sites. Our findings highlight the role of TLR polymorphisms in immune responses in the natural environment. G∗Power 3 software was used to calculate sample sizes from different infection groups,^38^ with the effect size assumed to be d = 0.5, the significance level α = 0.05, and the power of 1 - β = 0.8, the minimum sample size was calculated as 130 for the *Bartonella* group, 146 for the flea group, and 130 for the gamasid mite group. The sample size in this study was 150, which meets the sample size requirements.

Hamsters infested with fleas tend to be parasitized by gamasid mites, a phenomenon attributed to similar ecological requirements, host preferences, habits, and the host’s immune response to ectoparasites. Studies have shown that interactions between host species and biological communities or sampling periods similarly affect flea and mite diversity.^39^ Evolutionarily related host species often share similar parasitic compositions,^40^ reflecting co-evolutionary relationships where the host’s evolutionary history influences carried parasites. Additionally, ectoparasite bites promote blood-sucking behavior and inhibit host immune responses,^41^^,^^42^^,^^43^ potentially weakening defenses and facilitating secondary parasitism. We found a positive correlation between *Bartonella* infections and gamasid mite parasitism in striped hamsters, but not with flea parasitism. This difference may be due to mites serving as vectors for *Bartonella* transmission,^44^^,^^45^ which is more efficient than fleas. The host’s immune response to fleas may also differ from that to gamasid mites, affecting *Bartonella* transmission. Similar findings were observed in rats, where higher chigger mite loads correlated with increased *Bartonella* infection rates.^46^

TLRs play a crucial role in the innate immune system by recognizing various PAMPs^47^^,^^48^ and mounting pro-inflammatory responses to combat infectious agents, thereby maintaining immune homeostasis.^49^ However, the dysregulation of TLR activity due to genetic mutations can lead to immune-related pathologies.^27^^,^^50^^,^^51^^,^^52^^,^^53^ This study highlights the high polymorphism rates in six cell surface TLR genes, ranging from 1.68% to 3.49%, potentially resulting from diverse exogenous organism challenges. Notably, four key sites influencing *Bartonella* infection susceptibility were identified, with two on TLR1 and one each on TLR4 and TLR10. Common genotypes at three of these sites were protective against *Bartonella*, indicating that heterozygous or rare genotype carriers were more susceptible. Previous studies have revealed that specific sites on TLR1, TLR2, and TLR5 genes were significantly associated with the risk of *Bartonella* infection.^36^^,^^37^ We identified a polymorphic site in the TLR4 gene that was significantly associated with susceptibility to *Bartonella* infection. Notably, the lipopolysaccharide (LPS) of *Bartonella quintana* is a potent TLR4 antagonist that can specifically bind to TLR4, thereby inhibiting its activation.^54^^,^^55^ Based on the genetic association findings from this study, we hypothesize that polymorphisms in TLRs may influence the antagonistic effects of *Bartonella*’s LPS, thereby altering the activation status of the TLR4 signaling pathway and ultimately affecting the host’s immune defense against *Bartonella* infections. These sites may affect the ability of the host to recognize the components of *Bartonella*. Especially, mutations located on the TLR4 gene may directly affect the host’s sensitivity and responsiveness to LPS of *Bartonella*, leading to functional changes in the TLR signaling pathway and thus affecting the host’s immune defense ability against *Bartonella* infections. Previous studies have revealed that mutations in TLR4, a PRR, can modulate its pro-inflammatory response, and the distribution patterns of TLR4 polymorphisms arise from the combined effects of infectious pressure and genetic drift events, highlighting an evolutionary trade-off in innate immune genes between the benefits of antimicrobial defense and the risks of inflammatory pathology.^56^

In our study, we identified a total of 12 sites significantly influencing flea parasitism: 1 on the TLR4 gene, 5 on the TLR5 gene, and 6 on the TLR10 gene. The presence of heterozygous or rare genotypes at these sites was found to be beneficial for the host, preventing flea infection. Similarly, 11 sites were found to significantly affect gamasid mite parasitism, with 2 sites on the TLR5 gene, 1 on the TLR6 gene, and 7 on the TLR10 gene. These sites also exhibited similar protective effects when in the heterozygous or rare genotypes, shielding the host from gamasid mite infections. Notably, when one site on the TLR5 gene was a common genotype, it was beneficial for protecting the host from gamasid mite infections. Previous research suggested that ectoparasites can regulate the host’s TLR signaling pathway through their saliva secretions to influence the host’s immune response.^57^^,^^58^ Mutations in TLR genes may affect this process, impacting the host’s ability to recognize and respond to the components of ectoparasites' saliva, thereby influencing the risk of parasitism. The purpose of applying multiple statistical tests in this study is to explore the significant association between the genetic polymorphism of TLRs and host susceptibility to exogenous organism infections. We did not investigate the odds ratio of exogenous organism infections across multiple groups, and the use of multiple testing corrections may be too conservative, potentially leading to the exclusion of some polymorphic sites that could be significant. Therefore, we did not apply multiple testing corrections.

When a heterozygous genotype or rare genotype at this site was protective, hamsters with uninfected status exhibited greater genetic polymorphisms compared to those infected with exogenous organisms. This phenomenon may be attributed to natural selection favoring genotypes that enhance resistance to infections. As a result, these advantageous genotypes become more prevalent in the population, leading to high genetic diversity and adaptability. Conversely, the infected group shows lower genetic polymorphisms due to a lack of these favorable genotypes. For heterozygous and rare genotypes that were prone to exogenous organism infections in hosts, hosts carrying these genotypes were more susceptible to infections by exogenous organisms, thereby reducing their chances of survival and reproduction, resulting in a low frequency of these genotypes in uninfected host populations.

Our study performed an in-depth analysis of the specific localization of sites with significant effects on exogenous organism infections within the 3D structure of TLRs. Notably, all sites associated with *Bartonella* infections were situated in the extracellular domain, while approximately 50% of the sites associated with fleas and gamasid mite parasitism were found in the transmembrane and TIR domains. This distribution may reflect the distinct immune mechanisms triggered by bacteria and ectoparasites, as well as the functional differences among various regions of TLRs. A significant characteristic of the extracellular domain is the frequent occurrence of leucine-rich repeats (LRRs) sequences,^59^ which are crucial for recognizing PAMPs.^60^^,^^61^^,^^62^ The LRR domain plays a vital role in binding different pathogen components and triggering host immune responses.^22^^,^^63^^,^^64^ Our findings indicate that sites associated with *Bartonella* infections are concentrated in this domain, suggesting that mutations here could alter binding affinity and immune response intensity.^65^ Such changes might affect how TLRs recognize and bind to PAMPs present on *Bartonella*, thereby modulating the host’s immune system response to the bacteria. Conversely, about 25% and 36% of sites related to flea and gamasid mite parasitism, respectively, were located in the TIR domain. Fleas and gamasid mites, as ectoparasites, use their saliva to manipulate the host’s defense mechanisms, including immune and hemostatic systems.^41^^,^^42^ This manipulation likely involves the TIR domain, which initiates downstream signaling for inflammatory responses and adaptive immunity.^59^ Variations in this domain could alter downstream signal transduction,^66^ potentially benefiting the ectoparasites by interfering more effectively with the host’s immune response.

This study revealed significant associations between multiple polymorphic sites in host TLRs and susceptibility to *Bartonella* infection as well as ectoparasite infestation. Although the functional validation of these polymorphic sites remains pending, their biological plausibility is supported by existing literature. TLRs specifically recognize PAMPs through their LRR domains and activate the NF-κB pathway via MyD88-dependent signaling transduction mediated by TIR domains. Critical site mutations in TLRs may reduce recognition sensitivity to pathogenic PAMPs and impair the assembly efficiency of signaling complexes, thereby compromising their biological functions. Such functional defects could directly impact the timely release of innate immune effectors while attenuating the expression of co-stimulatory molecules and polarizing cytokines, ultimately leading to delayed or dysregulated adaptive immune responses.^19^^,^^67^ Previous studies have demonstrated that genetic variations in key TLR structural domains can modulate host defense mechanisms against pathogens by altering PAMPs recognition efficiency or signal transduction capacity, potentially undergoing balancing selection at microevolutionary scales driven by pathogen community dynamics.^36^^,^^37^ Despite current limitations in the functional validation of these TLR polymorphisms, the biological significance of these findings maintains a solid theoretical foundation based on existing functional investigations documented in prior literature.

### Limitations of the study

This study provides valuable insights into the role of cell surface TLR genes in immune regulation and resistance to infections. However, there are several limitations that need to be considered. Firstly, the study focused solely on striped hamsters, which limits the generalizability of the findings to other species. Secondly, while the study identified polymorphisms associated with susceptibility to *Bartonella* and ectoparasite infections, it did not investigate the functional mechanisms underlying these associations. Moreover, studies have shown that variations in host behavior significantly influence infection risk and transmission patterns.^68^ Consequently, this factor may act as a confounding variable affecting host susceptibility to pathogen infections. Currently, there is a lack of quantitative metrics for assessing behavioral differences among hosts in existing studies. The next step should be to quantify the behavioral differences of the host and further analyze the impact of TLR gene polymorphisms as a confounding factor on its infection with *Bartonella* and parasitism by ectoparasites. For future research, it is crucial to quantify these behavioral variations and conduct further analyses on how TLR gene polymorphisms influence *Bartonella* infections and the susceptibility to ectoparasites in the host. Despite these limitations, this research contributes to our understanding of the genetic basis of immune adaptation and highlights the potential for future investigations into the role of TLRs in infectious disease dynamics.

## Resource availability

### Lead contact

Further information and requests for resources and reagents should be directed to and will be fulfilled by the lead contact, Liang Lu (luliang@icdc.cn).

### Materials availability

This study did not generate new unique reagents.

### Data and code availability

• All datasets reported in this work are available from the lead contact on request.
• This article does not report original code.
• Any additional information required to reanalyze the data reported in this article is available from the lead contact upon request.

## Acknowledgments

This research was funded by the Major Program of the 10.13039/501100001809National Natural Science Foundation of China (NO.32090023), the Key R&D Program of Guangdong Province (NO.2022B1111030002) and the Operation of Public Health Emergency Response Mechanisms-Infectious Disease Prevention and Control (NO.102393220020020000029).

## Author contributions

Sample collection: L.L., G.C.L., N.Z., P.B.L., and Z.X.W.; experimental design: L.L., X.C.L., F.X.M., and Q.Y.L.; experiment conducted: X.C.L.; experimental assistance: Z.H.W., X.P.S., J.W., and Y.L.; data analysis: X.C.L.; writing—original draft: X.C.L.; writing—review and editing: L.L., X.C.L., Y.G.W., and Y.J.Y.

## Declaration of interests

The authors declare no competing interests.

## STAR★Methods

### Key resources table

**Table:** 

REAGENT or RESOURCE	SOURCE	IDENTIFIER
**Deposited data**		

Raw and analyzed data	This paper	N/A

**Software and algorithms**		

R	Version 4.3.2	https://www.r-project.org/
MEGA7	Version 7.0.26	https://www.megasoftware.net/
SnapGene	Version 6.0.2	https://www.snapgene.com/
PowerMarker	Version 3.25	https://doi.org/10.1093/bioinformatics/bti282
POPGENE	Version 1.32	https://sites.ualberta.ca/∼fyeh/popgene.html
SMART	N/A	http://smart.embl-heidelberg.de/
SWISS-MODEL	N/A	https://swissmodel.expasy.org/
PyMOL	Version 2.5.7	https://www.pymol.org/

### Experimental model and study participant details

#### Ethics statement

All animal experiments were conducted in accordance with the National Health guidelines for the welfare of experimental animals and with the approval of the Ethical Committee of the National Institute for Communicable Disease Control and Prevention, Chinese Center for Disease Control and Prevention (No. 2022-027).

#### Sample collection

Striped hamsters were captured using clamps and cages in the Inner Mongolia Autonomous Region during May, July and September of 2021–2022. The collection area included six sampling locations: Dongwuzhumuqin Banner, Xilingol League; Abaga Banner, Xilingol League; Xiwuzhumuqin Banner, Xilingol League; Xilinhot City, Xilingol League; Baiyinxile Ranch, Xilingol League; and Keerqin Left Middle Banner, Tongliao City (Figure S1). At each location, we placed 300 clamps and 100 cages baited with fresh peanuts. Each captured rodent was individually placed in a cloth bag, which was securely sealed to prevent parasites from escaping. Striped hamsters were identified among the captured rodents using morphological identification methods, and data such as collection time, location, sample gender, and weight were recorded. Parasites, including fleas and mites on the hamsters’ skin were collected in cryovials. Additionally, spleens were harvested, stored in liquid nitrogen, and subsequently transferred to a −80°C freezer for long-term storage.

### Method details

#### *Bartonella* detection

DNA was extracted from the spleens of the sampled striped hamsters using the reagent kit method. Quantitative real-time polymerase chain reaction (qPCR) was used to detect *Bartonella* infections.^69^ The qPCR system (20 μL) was as follows: 2 × Taq Pro HS Universal U+ Probe Master Mix, 10 μL; forward and reverse primers (10 μM) (ssrA-F, 5′-GCTATGGTAATAAATGGACAATGAAATAA-3′; ssrA-R, 5′-GCTTCTGTTGCCAGGTG-3′), 0.8 μL; probe (5′-FAM-ACCCCGCTTAAACCTGCGACG-3′-BHQ1), 0.8 μL; ddH2O, 4.6 μL; and DNA template, 3 μL. The reaction conditions were as follows: 95°C for 5 min, followed by 40 cycles of 95°C for 15 s and 60°C for 45 s. Both negative and positive controls were set up simultaneously. A cycle threshold (Ct) value of less than 35 was considered positive, indicating the presence of *Bartonella* infections.

#### Amplification of cell surface TLR genes

The longest CDSs of six cell surface TLR genes, including TLR1, TLR2, TLR4, TLR5, TLR6 and TLR10, were amplified from striped hamsters. Each CDS was divided into two segments for amplification. Primers were designed based on the reference gene sequences of these TLRs available on NCBI for the respective species. Detailed primer information was provided in Table S1, and the column named “Gene” in the table indicated the corresponding TLR gene. The PCR system (50 μL) was as follows: 2 × Phanta Max Master Mix, 25 μL; upstream and downstream primers (10 μM), 2μL; ddH2O, 19 μL; and DNA template, 2 μL. The reaction conditions were as follows: 95°C for 3 min, followed by 35 cycles of 95°C for 15 s, 60°C for 15 s, and 72°C for 1 min, then extended at 72°C for 5 min. The PCR products were analyzed by agarose gel electrophoresis, and positive bands were sequenced.

### Quantification and statistical analysis

#### Polymorphic sites in TLR genes

The two CDS fragments corresponding to each TLR gene were concatenated into a single continuous sequence, with the nucleotide positions numbered consecutively starting from the initial site of the first fragment. This facilitated precise identification and referencing of each nucleotide position throughout the combined CDS. MEGA7 software^70^ was used to align the sequences assembled from 150 samples, allowing for the identification of genomic locations and the number of sites in each TLR gene. The screening criteria for sites required the satisfaction of two conditions simultaneously: first, the frequency of base mutations in the site must be equal to or greater than 10% of the total samples; second, the site must be present in the top 10% of samples based on the number of samples exhibiting base mutations. We limited the number of sites to 10% of the total samples to ensure the feasibility and efficiency of the study. This criterion effectively narrowed the focus to sites likely to have the most significant impact. Finally, peak identification was used to assess and confirm the final base mutation status of the sites obtained through sequence alignment. SnapGene (https://www.snapgene.com/) software was used for peak plot comparison.

#### Statistical analysis

To investigate the relationship between genetic polymorphisms of the longest CDS and exogenous organism infections in striped hamsters, we used several statistical methods. To further explore the relationship between the sites of each TLR gene and exogenous organism infections, chi-square test, RF analysis and Boruta algorithm were used. RF is an ensemble learning method suitable for both classification and regression tasks, which calculates feature importance to evaluate the effect of variables on classification decisions.^71^^,^^72^^,^^73^ The Boruta algorithm extends RF by introducing shadow features to determine variable importance through their scores.^74^^,^^75^^,^^76^ These methods helped identify significant polymorphic sites affecting *Bartonella* and ectoparasite infections. GLM was used to analyze these significant sites individually, considering sites (0 for common genotypes, 1 for heterozygous genotypes, and 2 for rare genotypes) and influential gender or weight as independent variables, with *Bartonella*, flea and gamasid mite infections as dependent variables. Finally, the GBM model was used to rank the relative importance of each identified site in their respective TLR genes. GBM is an ensemble learning model used for classification and regression, constructed from multiple weak learning models,^77^^,^^78^ capable of demonstrating the importance ranking of each variable with high prediction accuracy. All statistical analyses were conducted using R4.3.2 software.

#### Genetic polymorphisms analysis

The PowerMarker 3.25 software^79^ was used to obtain information on PIC and heterozygosity. Additionally, the POPGENE 1.32 software (https://sites.ualberta.ca/∼fyeh/popgene.html) was used to calculate I and Nei. The SMART online tool^80^ was used to predict the important functional domains of the CDSs in six cell surface TLR genes. For 3D structure prediction of these TLR proteins, the SWISS-MODEL online tool^81^ was applied. PyMOL software^82^ was then used to draw the identified sites related to exogenous organism infections on the 3D structures of the proteins.
