# Supplementary material for: Cell surface Toll-like receptor polymorphisms influence Bartonella and ectoparasite infections in striped hamsters
Source: iScience. 2025 Jun 13;28(7):112883. doi: 10.1016/j.isci.2025.112883 (PMC12268665; doi:10.1016/j.isci.2025.112883)
Supplement: Document S1. Figures S1 and Table S1–S4 [file mmc1.pdf]

iScience, Volume *28*

Supplemental information

Cell surface Toll-like receptor polymorphisms influence *Bartonella* and ectoparasite infections in striped hamsters

Xinchang Lun, Yujuan Yue, Yiguan Wang, Guichang Li, Ning Zhao, Fengxia Meng, Qiyong Liu, Pengbo Liu, Zihao Wang, Zhenxu Wang, Xiuping Song, Jun Wang, Ying Liang, and Liang Lu

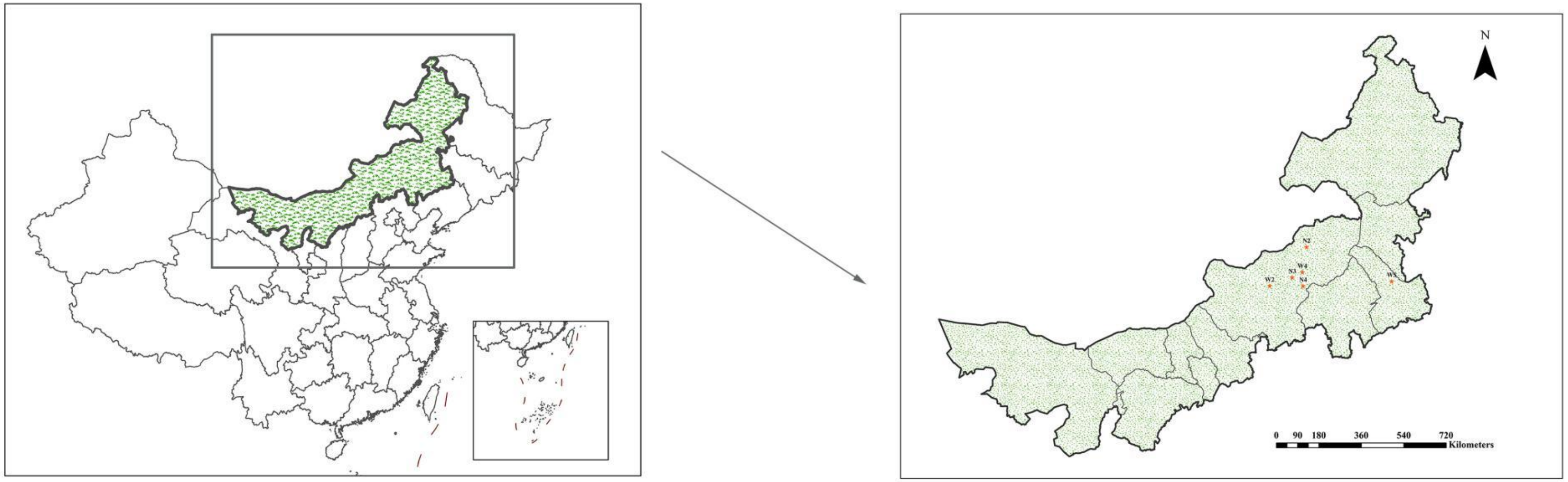

Figure S1. Schematic diagram of sampling location

(N2, Dongwuzhumuqin Banner, Xilingol League; N3, Xilinhot City, Xilingol League; N4, Baiyinxile Ranch, Xilingol League; W2, Abaga Banner, Xilingol League; W4, Xiwuzhumuqin Banner, Xilingol League; W5, Keerqin Left Middle Banner, Tongliao City).

Table S1. Primers for the longest CDS region in non-viral sensing TLR genes

Gene	Fragment	Primer	Sequence(5′-3′)	Product length (bp)
TLR1	TLR1_1	TLR1F1	5'-TCTGTACCCATGTTGAGATGC-3'	1396
		TLR1R1	5'-TCTGAAGACAGAGCCAGTAAG-3'	
	TLR1_2	TLR1F2	5'-TGTCTGCTGAGATGACATCG-3'	1244
		TLR1R2	5'-TCAGAACTGCCCAAAGCAGC-3'	
TLR2	TLR2_1	TLR2F1	5'-TGTACACGCACATTTGCAAG-3'	1407
		TLR2R1	5'-GAGTCAGGCATAGACTGAAAG-3'	
	TLR2_2	TLR2F2	5'-TTTGAAGAACGCAGCCTGTG-3'	1320
		TLR2R2	5'-AGGGTACAATTCTATGGCAC-3'	
TLR4	TLR4_1	TLR4F1	5'-GTCTCTTCACAATAACATGGTATATCACATC-3'	1318
		TLR4R1	5'-GATGTTGTCTTTGAAAGAATTGCCAG-3'	
	TLR4_2	TLR4F2	5'-CCTCAGCTTCAATGGTGTCATCAG-3'	1391
		TLR4R2	5'-GTCTACAACATAATCCATGGTGAGCTG-3'	
TLR5	TLR5_1	TLR5F1	5'-AGATCCCCAGGTCTGATGTG-3'	1422
		TLR5R1	5'-GGTTCTGATTCAGGATGAGAAACT-3'	
	TLR5_2	TLR5F2	5'-CCAGCTACATCCAGTTATCTGAGA-3'	1365
		TLR5R2	5'-TAATGGCTGGGCACATACAAC-3'	
TLR6	TLR6_1	TLR6F1	5'-CCACAATGCTCATCAGAGTTCAAG-3'	1334
		TLR6R1	5'-CCAAGGCTGCTCCACCTTAAAG-3'	
	TLR6_2	TLR6F2	5'-CCCTCATATGCATGAGAGACCAAG-3'	1394
		TLR6R2	5'-CACTTAAAATCCTGGGAAATGGAAGC-3'	
TLR10	TLR10_1	TLR10F1	5'-CTCACCCTTCTGTGCTGTTTTCCA-3'	1359
		TLR10R1	5'-GGTCAAGGTGTCTGGCCACA-3'	
	TLR10_2	TLR10F2	5'-GGTGAGTGTCTTTGCCAACAG-3'	1306
		TLR10R2	5'-AACAGTGATGGTGGTCCCT-3'	

The reference sequences of the coding sequence of TLR1, TLR2, TLR4, TLR5, TLR6 and TLR10 genes were obtained from NCBI, with the following accession numbers: XM_007607728.4, XM_027434433.2, XM_035460226.1, XM_027435517.2, XM_003515801.5 and XM_035438383.1. Among them, the primer sequences for TLR1, TLR2, TLR4, TLR5 and TLR10 genes were designed in forward directions, while the primer sequence for TLR6 gene was designed in a reverse direction.

Table S2. Genetic polymorphisms associated with *Bartonella* infections (P<0.05)

TLRs	Sites	Genotype	Uninfected	Infected	χ^2	P-value
TLR1	1_1793	CC	26(40.0%)	47(55.3%)	3.449	0.063
		CT	33(50.8%)	28(32.9%)	4.852	0.028
		TT	6(9.2%)	10(11.8%)	0.248	0.618
	1_1492	CC	26(40.0%)	20(23.5%)	4.699	0.030
		CT	30(46.2%)	47(55.3%)	1.232	0.267
		TT	9(13.8%)	18(21.2%)	1.341	0.247
	1_1331	GG	54(83.1%)	81(95.3%)	6.109	0.013
		GA	11(16.9%)	4(4.7%)	6.109	0.013
TLR4	4_36	AA	62(95.4%)	71(83.5%)	5.152	0.023
		AG	3(4.6%)	14(16.5%)	5.152	0.023
TLR6	6_308	GG	30(46.2%)	35(41.2%)	0.372	0.542
		GA	25(38.5%)	46(54.1%)	3.622	0.057
		AA	10(15.4%)	4(4.7%)	4.964	0.026
	6_2187	GG	34(52.3%)	38(44.7%)	0.853	0.356
		GA	22(33.8%)	44(51.8%)	4.800	0.028
		AA	9(13.8%)	3(3.5%)	5.327	0.021
	6_2151	CC	32(49.2%)	40(47.1%)	0.070	0.792
		CT	25(38.5%)	43(50.6%)	2.186	0.139
		TT	8(12.3%)	2(2.4%)	-	0.021
TLR10	10_438	CC	61(93.8%)	70(82.4%)	4.398	0.036
		CT	4(6.2%)	15(17.6%)	4.398	0.036

Table S3. Genetic polymorphisms associated with flea infections ($P<0.05$)

TLRs	Sites	Genotype	Uninfected	Infected	χ^2	*P*-value
TLR4	4_1441	GG	59(58.4%)	37(75.5%)	4.185	0.041
		GT	39(38.6%)	12(24.5%)	2.933	0.087
		TT	3(3.0%)	0(0.0%)	-	0.551
	4_1708	CC	27(26.7%)	11(22.4%)	0.320	0.572
		CT	46(45.5%)	31(63.3%)	4.147	0.042
		TT	28(27.7%)	7(14.3%)	3.330	0.068
TLR5	5_25	TT	37(36.6%)	29(59.2%)	6.809	0.009
		CT	48(47.5%)	15(30.6%)	3.874	0.049
		CC	16(15.8%)	5(10.2%)	0.871	0.351
	5_99	AA	37(36.6%)	28(57.1%)	5.652	0.017
		AG	54(53.5%)	19(38.8%)	2.850	0.091
		GG	10(9.9%)	2(4.1%)	-	0.338
	5_91	GG	41(40.6%)	31(63.3%)	6.794	0.009
		GA	45(44.6%)	16(32.7%)	1.937	0.164
		AA	15(14.9%)	2(4.1%)	3.808	0.051
	5_639	CC	54(53.5%)	36(73.5%)	5.501	0.019
		CG/CT	41(40.6%)	12(24.5%)	3.745	0.053
		GG	6(5.9%)	1(2.0%)	-	0.428
	5_614	AA	61(60.4%)	40(81.6%)	6.765	0.009
		AG	35(34.7%)	9(18.4%)	4.222	0.040
		GG	5(5.0%)	0(0.0%)	-	0.173
TLR10	10_1110	GG	82(81.2%)	48(98.0%)	8.031	0.005
		GT	18(17.8%)	1(2.0%)	7.428	0.006
		TT	1(1.0%)	0(0.0%)	-	1.000
	10_1801	CC	82(81.2%)	48(98.0%)	8.031	0.005
		CT	18(17.8%)	1(2.0%)	7.428	0.006
		TT	1(1.0%)	0(0.0%)	-	1
	10_1807	CC	82(81.2%)	48(98.0%)	8.031	0.005
		CT	18(17.8%)	1(2.0%)	7.428	0.006
		TT	1(1.0%)	0(0.0%)	-	1
	10_1899	AA	82(81.2%)	48(98.0%)	8.031	0.005
		AG	18(17.8%)	1(2.0%)	7.428	0.006
		GG	1(1.0%)	0(0.0%)	-	1
	10_2332	AA	82(81.2%)	48(98.0%)	8.031	0.005
		AT	18(17.8%)	1(2.0%)	7.428	0.006
		TT	1(1.0%)	0(0.0%)	-	1
	10_2333	TT	82(81.2%)	48(98.0%)	8.031	0.005
		TC	18(17.8%)	1(2.0%)	7.428	0.006
		CC	1(1.0%)	0(0.0%)	-	1

Table S4. Genetic polymorphisms associated with gamasid mite infections (P<0.05)

TLRs	Sites	Genotype	Uninfected	Infected	χ^2	P-value
TLR1	1_174	CC	53(66.3%)	47(74.6%)	1.170	0.280
		CT	25(31.3%)	9(14.3%)	5.597	0.018
		TT	2(2.5%)	7(11.1%)	-	0.043
TLR2	2_728	AA	32(40.0%)	28(44.4%)	0.286	0.593
		AG	27(33.8%)	27(42.9%)	1.244	0.265
		GG	21(26.3%)	8(12.7%)	4.004	0.045
TLR5	5_2322	GG	37(46.3%)	16(25.4%)	6.570	0.010
		GA	37(46.3%)	40(63.5%)	4.216	0.040
		AA	6(7.5%)	7(11.1%)	0.556	0.456
	5_91	GG	31(38.8%)	35(55.6%)	4.005	0.045
		GA	41(51.3%)	19(30.2%)	6.438	0.011
		AA	8(10.0%)	9(14.3%)	0.618	0.432
	5_1500	CC	52(65.0%)	53(84.1%)	6.608	0.010
		CA/CT	27(33.8%)	10(15.9%)	5.873	0.015
		AA	1(1.3%)	0(0.0%)	-	1.000
	5_180	TT	59(73.8%)	56(88.9%)	5.130	0.024
		TG	19(23.8%)	7(11.1%)	3.785	0.052
		GG	2(2.5%)	0(0.0%)	-	0.504
TLR6	6_159	CC	44(55.0%)	46(73.0%)	4.904	0.027
		CT	32(40.0%)	15(23.8%)	4.187	0.041
		TT	4(5.0%)	2(3.2%)	-	0.694
TLR10	10_806	CC	62(77.5%)	59(93.7%)	7.062	0.008
		CT	16(20.0%)	4(6.3%)	5.459	0.019
		TT	2(2.5%)	0(0.0%)	-	0.504
	10_1110	GG	63(78.8%)	61(96.8%)	9.995	0.002
		GT	16(20.0%)	2(3.2%)	9.068	0.003
		TT	1(1.3%)	0(0.0%)	-	1.000
	10_1801	CC	63(78.8%)	61(96.8%)	9.995	0.002
		CT	16(20.0%)	2(3.2%)	9.068	0.003
		TT	1(1.3%)	0(0.0%)	-	1
	10_1807	CC	63(78.8%)	61(96.8%)	9.995	0.002
		CT	16(20.0%)	2(3.2%)	9.068	0.003
		TT	1(1.3%)	0(0.0%)	-	1
	10_1899	AA	63(78.8%)	61(96.8%)	9.995	0.002
		AG	16(20.0%)	2(3.2%)	9.068	0.003
		GG	1(1.3%)	0(0.0%)	-	1
	10_2332	AA	63(78.8%)	61(96.8%)	9.995	0.002
		AT	16(20.0%)	2(3.2%)	9.068	0.003
		TT	1(1.3%)	0(0.0%)	-	1
	10_2333	TT	63(78.8%)	61(96.8%)	9.995	0.002
		TC	16(20.0%)	2(3.2%)	9.068	0.003
		CC	1(1.3%)	0(0.0%)	-	1
